# A hormone-dependent tRNA half promotes cell cycle progression via destabilization of p21 mRNA

**DOI:** 10.1371/journal.pbio.3003194

**Published:** 2025-06-05

**Authors:** Takuya Kawamura, Megumi Shigematsu, Yohei Kirino

**Affiliations:** 1 Computational Medicine Center, Sidney Kimmel Medical College, Thomas Jefferson University, Philadelphia, Pennsylvania, United States of America; 2 Department of Biochemistry and Molecular Biology, Sidney Kimmel Medical College, Thomas Jefferson University, Philadelphia, Pennsylvania, United States of America; Johns Hopkins University, UNITED STATES OF AMERICA

## Abstract

tRNA halves are among the most abundant short non-coding RNAs in the cellular transcriptome. Here we report that in androgen receptor-positive LNCaP prostate cancer cells, the hormone-dependent 5′-tRNA^LysCUU^ half promoted cell proliferation by facilitating cell cycle progression. Global mRNA profiling upon the 5′-tRNA^LysCUU^ half depletion revealed that the mRNA of p21, a negative regulator of the cell cycle, is post-transcriptionally destabilized via a 5′-tRNA^LysCUU^ half-driven mechanism. YBX1, identified as a protein interacting with 5′-tRNA^LysCUU^ half in the cytosol, was shown to stabilize p21 mRNA. Specific sequences resembling the 5′-tRNA^LysCUU^ half, located in the 3′-UTR of p21 mRNA and termed LL588, were identified as the binding site for YBX1 and are required for p21 mRNA stability. In vitro binding assays demonstrated that the 5′-tRNA^LysCUU^ half is capable of displacing YBX1 from LL588. Collectively, our findings suggest that the 5′-tRNA^LysCUU^ half directly binds to and displaces YBX1 from p21 mRNA, leading to the destabilization of p21 mRNA and the promotion of cell cycle progression in hormone-dependent cancers. Our study illuminates the role of tRNA halves in regulating mRNA stability and suggests that this may be part of broader regulatory networks affecting mRNA levels, orchestrated by various tRNA halves and their interacting proteins.

## Abstract 2

Androgen receptor-positive prostate cancer cells abundantly express hormone-dependent tRNA halves, but their functions are unclear. These authors show that the 5′-tRNALysCUU half promotes cell cycle progression by destabilizing the mRNA of p21, a negative regulator, suggesting a general role for these molecules.

## Introduction

Short non-coding RNAs (sncRNAs) constitute a heterogeneous group of RNA molecules, including microRNAs (miRNAs) and transfer RNA (tRNA)-derived sncRNAs, and their aberrant expression is involved in the onset and progression of many human diseases, including cancers. The most abundant class of tRNA-derived sncRNAs is tRNA halves, which, in mammalian cells, are produced by the endoribonucleolytic cleavage of tRNA anticodon-loop by angiogenin (ANG), a member of the RNase A superfamily [1–3]. The ANG-catalyzed tRNA cleavage is triggered by diverse cellular stimuli such as oxidative stress, other forms of stress, and mycobacterial infections [1,2,4]. The resultant accumulation of tRNA halves has been shown to regulate translation, foster stress granule formation, and promote immune response [1,2,4]. However, in contrast to miRNA molecules which are known to possess common binding proteins (i.e., Argonaute proteins) and have well-defined mechanisms behind their regulatory roles in gene expression, knowledge remains limited regarding the binding partners of tRNA halves and the molecular mechanisms underlying their function.

Our previous screenings of over 90 cancer cell lines revealed sex hormone-dependent expression of tRNA half molecules [5]. In estrogen receptor (ER)-positive breast cancer cells and androgen receptor (AR)-positive prostate cancer cells, the sex hormone signaling pathways induce ANG-mediated cleavage of aminoacylated mature tRNAs, leading to abundant accumulations of tRNA halves. cP-RNA-seq [5,6] identified only eight cytoplasmic tRNA isoacceptors, such as tRNA^LysCUU^, tRNA^HisGUG^, and tRNA^AspGUC^, as major sources for these hormone-dependent tRNA halves. While functional studies in AR-positive LNCaP prostate cancer cells indicated that the hormone-dependent 5′-tRNA halves, but not the 3′-tRNA halves, promote cell proliferation [5], the underlying mechanism for this remained elusive. Here, by analyzing mRNA expression profiles upon 5′-tRNA half depletion, we identified p21, a cell cycle inhibitor, whose mRNA is destabilized by the 5′-tRNA half. This destabilization is via the 5′-tRNA half-mediated displacement of the mRNA-stabilizing protein, which, in the absence of the 5′-tRNA half, interacts with tRNA half-like sequences encoded in the 3′-untranslated region (UTR) of the mRNA for stabilization. Our findings elucidate the tRNA half-mediated regulation of mRNA stability, and this first example might imply widespread regulatory networks of mRNA levels orchestrated by tRNA halves—one of the predominant sncRNA species—and their interacting proteins.

## Results and discussion

### 5′-tRNA^LysCUU^ half promotes cell cycle progression

**Fig 1:**
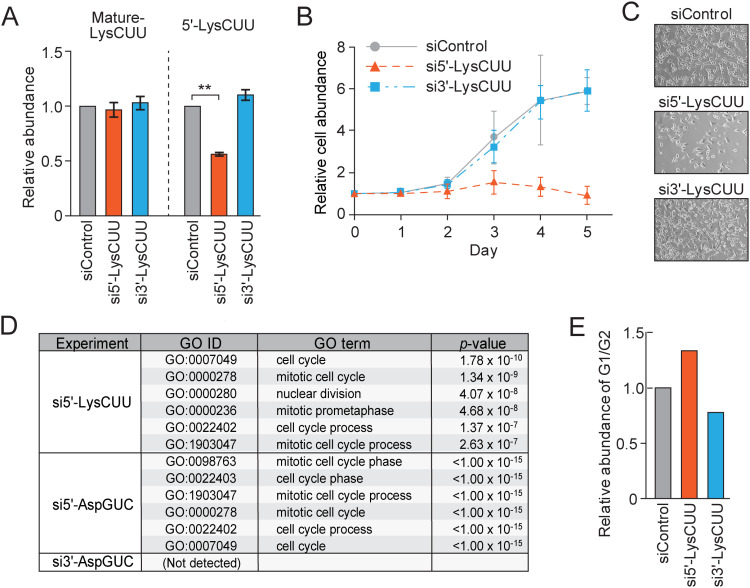
Knockdown of the 5′-tRNA^LysCUU^ half suppresses cell cycle progression. **(A)** Quantification of mature tRNA^LysCUU^ and its 5′-half by RT-qPCR and TaqMan RT-qPCR, respectively, upon transfection of the indicated siRNAs. For all bar graphs in the present study, error bars indicate mean ± SD of triplicate measurements (*P < 0.05, **P < 0.01, and ***P < 0.001; two-tailed *t*-Test). **(B)** The relative abundance of LNCaP cells af*t*er transfection of the indicated siRNAs. Abundance on the day of transfection was set at 1. Each dataset represents the average of three independent experiments with error bars showing the SD. **(C)** Representative images of the cells upon the siRNA transfection (Day 3). **(D)** The six most enriched GO terms upon KDs of tRNA halves, revealed using Panther enrichment analysis. No GO term with significant changes (*P* < 0.001) was detected upon the KD of 3′-tRNA^AspGUC^ half. **(E)** Cell cycle analyses using flow cytometry upon KDs of the indicated tRNA haves. The data underlying the graphs can be found in S1 Data.

To elucidate the molecular functions of sex hormone-dependent tRNA halves, we employed siRNA-mediated knockdown (KD) of tRNA halves in AR-positive LNCaP prostate cancer cells. We focused on the 5′-tRNA^LysCUU^ half, ranging from nucleotide position (np) 1−34, referred to as tDR-1:34-Lys-CTT-1-M2 by tDRnamer [7] and as tRF-34-PSQP4PW3FJIKE5 by MINTBase [8] (S1 Table). This tRNA half was selected due to its identification as the most abundant sex hormone-dependent tRNA half species [5] and its prominence among tRNA half species in various human cells and mouse tissues [4,9,10]. As previously reported [5], the KD of the 5′-tRNA^LysCUU^ half specifically reduced its levels without impacting the mature tRNA^LysCUU^ (Fig 1A). The 5′-tRNA^LysCUU^ half KD hindered cell growth (Fig 1B, 1C), while the KD of the 3′-tRNA^LysCUU^ half (S1 Table) did not affect the cell growth rate, confirming the role of 5′-tRNA halves in promoting cell proliferation. To uncover the mechanism behind this, we conducted an mRNA microarray analysis to discern gene expression alterations following KDs of 5′- or 3′-tRNA halves compared to control KD. PANTHER enrichment analysis of a total of 19,699 genes commonly identified cell cycle-related GO terms as the most altered biological process following the KDs of 5′-tRNA^LysCUU^ half and 5′-tRNA^AspGUC^ half (Fig 1D), whereas no biological process showed significant (*P* < 0.001) changes upon the KD of 3′-tRNA^AspGUC^ half. In consistent with these observations, propidium iodide (PI) flow cytometric analysis revealed a higher proportion of the cells in the G1 phase upon 5′-tRNA^LysCUU^ half KD but not upon 3′-tRNA^LysCUU^ half KD (Fig 1E), indicating that the cell cycle progression from G1 to S phase was stalled upon the 5′-tRNA^LysCUU^ half KD. Collectively, these results suggest that the 5′-tRNA^LysCUU^ half propels cell cycle progression by modulating cell cycle-related transcriptome.

### 5′-tRNA^LysCUU^ half post-transcriptionally destabilizes p21 mRNA

**Fig 2:**
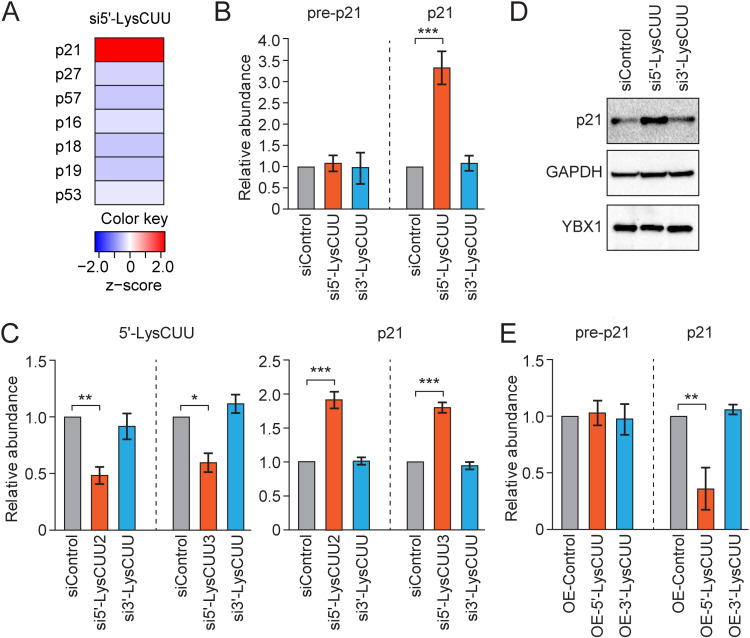
The p21 mRNA is destabilized by the 5′-tRNA^LysCUU^ half. **(A)** Changes of the mRNA levels of CDK inhibitors and p53 upon 5′-tRNA^LysCUU^ half KD from microarray analysis. **(B, C,** **E)** Total RNAs extracted from 5′- or 3′-tRNA^LysCUU^ half KD **(B and C)** or OE **(E)** cells were subjected to RT-qPCR for the quantification of p21 and pre-p21, and to TaqMan RT-qPCR for the quantification of 5′-tRNA^LysCUU^ half. As a negative control, control siRNA or control oligo with Rluc sequences was used for KD and OE experiments, respectively. For all bar graphs in the present study, error bars indicate mean ± SD of triplicate measurements (*P < 0.05, **P < 0.01, and ***P < 0.001; two-tailed *t*-Test). **(D)** Lysates from the cells were subjected to western blots for p21 protein. Raw images are located in Sl Raw images. The data underlying the graphs can be found in S1 Data.

When we analyzed the expression of individual cell cycle regulators, p21 mRNA, which encodes cyclin-dependent kinase (CDK) inhibitor 1, emerged as the most upregulated mRNA upon 5′-tRNA^LysCUU^ half KD (Figs 2A, S1). The mRNAs of other CDK inhibitors, such as p27, p57, p16, and p18, did not show significant alterations. Given that p21 binds to the cyclin E/CDK2, a G1/S cyclin complex, thereby inhibiting its function [11], the upregulation of p21 may underlie the observed G1 cell cycle stall and cell growth impairment induced by 5′-tRNA^LysCUU^ half KD. RT-qPCR verified the consistent elevation in p21 mRNA levels upon 5′-tRNA^LysCUU^ half KD (Fig 2B), a pattern not observed with 3′-tRNA^LysCUU^ half KD. Two additional siRNAs targeting the 5′-tRNA^LysCUU^ half consistently upregulated the levels of p21 mRNA, helping to rule out the possibility of off-target effects from the siRNAs (Fig 2C). The levels of precursor p21 (pre-p21) mRNA, which contains introns, remained unaffected by the 5′-tRNA half KD (Fig 2B). This aligns with our observation that the mRNA levels of p53, the primary transcription factor for *p21*, also remained unchanged (Fig 2A). These results suggest that the p21 mRNA upregulation does not stem from increased transcription of the *p21* gene but from enhanced mRNA stability at post-transcriptional levels. We confirmed that the increased levels of p21 mRNA indeed leads to enhanced p21 protein expression (Fig 2D). Contrary to the KD experiment results, overexpression (OE) of 5′-tRNA^LysCUU^ half in turn reduced p21 mRNA levels without affecting pre-p21 mRNA (Fig 2E), while 3′-tRNA^LysCUU^ half OE had no effect on both p21 and pre-p21 mRNAs. Taken together, these results suggest the role of 5′-tRNA^LysCUU^ half in the post-transcriptional destabilization of p21 mRNA, which could be a key mechanism driving the 5′-tRNA half-mediated promotion of cell cycle progression and cell proliferation.

### YBX1, a 5′-tRNA^LysCUU^ half interacting protein, stabilizes p21 mRNA and is involved in 5′-tRNA^LysCUU^ half-mediated promotion of cell proliferation

**Fig 3:**
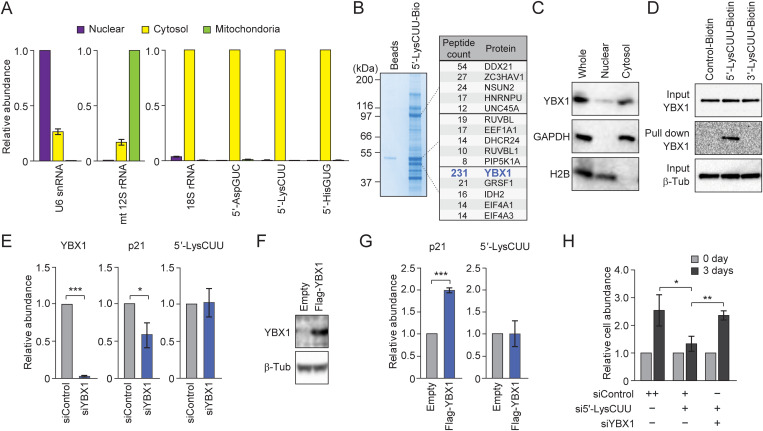
The p21 mRNA is stabilized by YBX1, a protein that interacts with 5′-tRNA^LysCUU^ half. **(A)** Nuclear, cytosolic, and mitochondrial fractions of LNCaP cells were subjected to RT-qPCR for the quantification of U6 snRNA, mitochondrial 12S rRNA, and 18S rRNA, as well as to TaqMan RT-qPCR for the quantification of 5′-tRNA halves. U6 snRNA, mitochondrial 12S rRNA, and 18S rRNA served as controls for nuclear, mitochondrial, and cytosolic RNA, respectively. **(B)** Proteins interacting with 5′-tRNA^LysCUU^ half from pull-down experiments were developed by SDS–PAGE. Three prominent bands were subjected to mass-spec protein identification. The identified Top 5 proteins are shown. **(C)** The total protein lysate (Whole), nuclear, and cytosolic fractions of LNCaP cells were subjected to western blots. H2B and GAPDH were used as nuclear and cytosolic protein controls, respectively. **(D)** Cytosolic lysates from pull-down experiments using biotinylated control RNA or 5′-/3′-tRNA^LysCUU^ half were subjected to western blot for YBX1. Input lysates before pull-down experiments were also analyzed as a control. **(E, G)** Total RNAs extracted from YBX1 KD **(E)** or OE **(G)** cells were subjected to RT-qPCR/TaqMan RT-qPCR for quantification of YBX1 mRNA, p21 mRNA, and 5′-tRNA^LysCUU^ half. For all bar graphs in the present study, error bars indicate mean ± SD of triplicate measurements (*P < 0.05, **P < 0.01, and ***P < 0.001; two-tailed *t*-Test). **(F)** Lysates from the YBX1 OE cells were subjected to western blots. **(H)** The relative abundance of the cells after transfection of the indicated siRNAs. To ensure consistency, the total amounts of siRNA were adjusted to be identical. The condition transfected only with control siRNA contained double the amount of the control siRNA, as the other two conditions contained two species of siRNAs. Abundance on the day of transfection was set at 1. Raw images of **B, C, D, and F** are located in S1 Raw images. The data underlying the graphs can be found in S1 Data.

Because we confirmed the predominant localization of 5′-tRNA halves in the cytosol of LNCaP cells (Fig 3A), we aimed to identify cytosolic 5′-tRNA^LysCUU^ half-interacting proteins that could function in the 5′-tRNA^LysCUU^ half-mediated regulation of p21 mRNA stability. Pull-down experiments using biotinylated 5′-tRNA^LysCUU^ half with LNCaP cell lysate, followed by mass-spec analyses, identified Y-box binding protein 1 (YBX1) as one of the most major interacting proteins of the 5′-tRNA^LysCUU^ half (Fig 3B). YBX1 is a multifunctional nucleic acid-binding protein implicated in diverse processes, such as mRNA stability, DNA repair, and cancer progression [12], and has been shown as a tRNA half-interacting protein [13]. Though both nuclear and cytosolic roles of YBX1 have been demonstrated [12], its primary localization in LNCaP cells is in the cytosol (Fig 3C). Further pull-down experiments using the cytosolic fraction confirmed that YBX1 specifically interacts with 5′-tRNA^LysCUU^ half but not with 3′-tRNA^LysCUU^ half or control RNA oligos (Fig 3D). Strikingly, siRNA-mediated KD of YBX1 led to a decrease in p21 mRNA levels (Fig 3E), while OE of YBX1 increased p21 mRNA levels (Fig 3F–3G). The levels of 5′-tRNA^LysCUU^ half remained unchanged by either KD or OE of YBX1 (Fig 3E, 3G), suggesting that YBX1, while not influencing 5′-tRNA^LysCUU^ half levels, does stabilize p21 mRNA. To confirm the involvement of YBX1 in the 5′-tRNA^LysCUU^ half-mediated pathway, we performed simultaneous KDs of YBX1 and the 5′-tRNA^LysCUU^ half in our cell proliferation assay. While the 5′-tRNA^LysCUU^ half KD alone significantly impaired cell growth (as also shown in Fig 1B, 1C), the impairment was rescued by the concurrent KD of YBX1 (Fig 3H), indicating the requirement of YBX1 in the 5′-tRNA^LysCUU^ half-driven promotion of cell proliferation.

### 5′-tRNA^LysCUU^ half displaces YBX1 from 5′-tRNA^LysCUU^ half-like sequences in 3′-UTR of p21 mRNA

**Fig 4:**
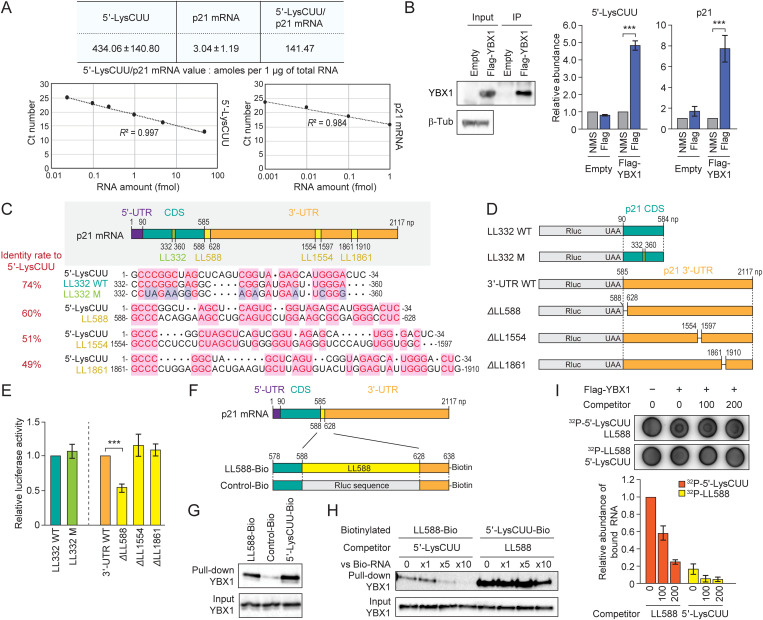
5′-tRNA^LysCUU^ half-mediated displacement of YBX1 protein from LL588 in the 3′-UTR of p21 mRNA. **(A)** Absolute abundance of 5′-tRNA^LysCUU^ half and p21 mRNA was quantified by TaqMan RT-qPCR and RT-qPCR, respectively, and calculated based on standard curves from quantification of various amounts of targeted RNAs. Averages of three independent experiments with SD values and the ratio of the abundance of 5′-tRNA^LysCUU^ half versus p21 mRNA are shown. **(B)** UV-crosslinking and immunoprecipitation of YBX1, followed by western blot detection of the purified YBX1 (left) and TaqMan RT-qPCR/RT-qPCR detection of the 5′-tRNA^LysCUU^ half and p21 mRNA (right). For all bar graphs in the present study, error bars indicate mean ± SD of triplicate measurements (*P < 0.05, **P < 0.01, and ***P < 0.001; two-tailed *t* Test). **(C)** The positions and sequences of four LLs in p21 mRNA. Iden*t*ity rates to the sequences of the 5′-tRNA^LysCUU^ half are shown. **(D)** Reporter constructs for luciferase assays to analyze the influence of LLs on mRNA stability. The CDS (with or without silent mutations in LL332) or the 3′-UTR (with or without LL588, LL1554, or LL1861) of p21 mRNA was attached to the downstream of Rluc mRNA. **(E)** Luciferase reporter assay. The constructs were transfected into LNCaP cells, followed by analysis of luciferase activity. **(F)** Biotinylated RNAs for pull-down experiments. Biotinylated p21 mRNA fragment of positions 578–638 (LL588-Bio: LL588 sequences with additional 10 nucleotides of 5′- and 3′-sequences) and biotinylated control RNA (Control-Bio: LL588 sequences in LL588-Bio were replaced with Rluc sequences) were used. **(G)** Pull-down experiments. The beads from the pull-down experiments using the indicated biotinylated RNAs were subjected to western blot for YBX1. Input lysates before pull-down experiments were also analyzed as a control. **(H)** Pull-down competition assay in which the beads from pull-down experiments using 5′-LysCUU-Bio or LL588-Bio were incubated with the competitor LL588 RNA or 5′-tRNA^LysCUU^ half, respectively, followed by washing and western blot for YBX1 protein. Input lysates before pull-down experiments were also analyzed as a control. **(I)** Competition assay using DRaCALA. Recombinant YBX1 protein was incubated with either ^32^P-labeled 5′-tRNA^LysCUU^ half and LL588 or ^32^P-labeled LL588 and 5′-tRNA^LysCUU^ half. Unlabeled RNAs were used as competitors at concentrations of 100 μM or 200 μM. The bar graph represents the relative amounts of the indicated ^32^P-IabeIed RNAs bound to YBX1 Raw images of **B, G, H, and I** are located in S1 Raw images. The data underlying the graphs can be found in S1 Data.

Considering the interaction between YBX1 and 5′-tRNA^LysCUU^ half, as well as the observed increase or decrease in the stability of p21 mRNA upon KD of 5′-tRNA^LysCUU^ half or YBX1, respectively, we reasoned that the 5′-tRNA^LysCUU^ half and p21 mRNA compete for binding to YBX1. We hypothesized that, in the absence of 5′-tRNA^LysCUU^ half, YBX1 binds to the p21 mRNA to stabilize it. However, when the 5′-tRNA^LysCUU^ half is abundantly accumulated by sex hormone signaling pathways, it sequesters YBX1 through direct interaction, thereby releasing YBX1 from its binding to p21 mRNA and leading to the mRNA’s destabilization. The regulatory mechanism of mRNA levels involving YBX1 and their interacting tRNA-derived sncRNAs was first identified by Tavazoie’s group [14]; however, no studies have yet investigated the involvement of tRNA halves, the most abundant tRNA-derived sncRNAs, in these mechanisms. We explored the quantitative relationship between the 5′-tRNA^LysCUU^ half and p21 mRNA by determining the absolute amounts of both molecules. The calculations of their abundances were based on standard curves generated from various amounts of the two RNAs, which showed excellent linearity between input amounts and amplification signals (Fig 4A). As a result, the abundance of the 5′-tRNA^LysCUU^ half is significantly higher (over 140-fold) compared to that of p21 mRNA (Fig 4A).

Through UV-crosslinking and immunoprecipitation of YBX1, we observed direct interactions between YBX1 and both the 5′-tRNA^LysCUU^ half and p21 mRNA (Fig 4B). Because YBX1 binds to the 5′-tRNA^LysCUU^ half but not to the 3′-tRNA^LysCUU^ half (Fig 3D), we searched for sequences in p21 mRNA that resemble the 5′-tRNA^LysCUU^ half as potential binding sites for YBX1. We identified four such sequences, referred to as 5′-tRNA^LysCUU^ half-Like sequences (LLs), starting from np 332, 588, 1,554, and 1861 (LL332, LL588, LL1554, and LL1861, respectively) (Fig 4C). The sequence identity rates to the 5′-tRNA^LysCUU^ half were 74%, 60%, 51%, and 49%, respectively. LL332 is located within the coding sequence (CDS) of p21 mRNA, while the other three LLs are in the 3′-UTR.

To analyze the influence of these LLs on p21 mRNA stability, we employed luciferase reporter constructs (Fig 4D). Silent mutations in LL332, which reduced its identity rates to 37%, had no effect on luciferase activity (Fig 4E). However, among the LLs in the 3′-UTR, the loss of LL588 led to clear reduction in luciferase activity (Fig 4E), suggesting that LL588 is an important sequence for enhancing mRNA stability. The deletion of LL1554 and LL1861, which have lower sequence identity rates to the 5′-tRNA^LysCUU^ half compared to LL588, did not affect luciferase activity. Moreover, the luciferase assay using cells with KD or OE of the 5′-tRNA^LysCUU^ half showed no effect of the tRNA half on the LL588-lacking construct, whereas the wild-type or LL1554-lacking construct showed increased or decreased activity in KD or OE cells, respectively (S2 Fig). These data suggest the involvement of the 5′-tRNA^LysCUU^ half in LL588-derived mRNA stability.

To investigate whether YBX1 binds to the LL588 sequences, we performed pull-down experiments using biotinylated LL588 RNA and control RNA with Renilla luciferase (Rluc) sequences (Fig 4F), as well as the 5′-tRNA^LysCUU^ half as a positive control, using cytosolic lysate of LNCaP cells. Subsequent western blot analysis revealed an interaction between LL588 and YBX1, although the abundance of the pulled down YBX1 protein suggests that its interaction with 5′-tRNA^LysCUU^ half is stronger than that with LL588 (Fig 4G). To explore whether 5′-tRNA^LysCUU^ half could displace YBX1 from LL588, we conducted a pull-down competition assay in which the pull-down beads bound to either LL588 or 5′-tRNA^LysCUU^ half were mixed with a competitor RNA – either 5′-tRNA^LysCUU^ half or LL588, respectively. As shown in Fig 4H, a 5-fold greater abundance of 5′-tRNA^LysCUU^ half compared to LL588 RNA effectively trapped most of the YBX1 protein that had been pulled down with LL588. A 10-fold greater abundance completely displaced YBX1 from LL588. This suggests that the >140-fold greater abundance of 5′-tRNA^LysCUU^ half to p21 mRNA (Fig 4A) is sufficient to trap YBX1 protein in LNCaP cells. On the other hand, even a 10-fold greater abundance of LL588 RNA could not completely capture YBX1 from the 5′-tRNA^LysCUU^ half, confirming the stronger affinity of YBX1 for the 5′-tRNA^LysCUU^ half over LL588. To further validate these results, we performed a Differential Radial Capillary Action of Ligand Assay (DRaCALA) [15] using recombinant YBX1 protein purified from HEK293T cells (S3A Fig). As expected, YBX1 protein interacted more strongly with the 5′-tRNA^LysCUU^ half than with LL588, and no binding was detected with the 3′-tRNA^LysCUU^ half (S3B Fig). The competition assay using DRaCALA, where ^32^P-labeled 5′-tRNA^LysCUU^ half or LL588 was incubated with unlabeled competitors (LL588 or 5′-tRNA^LysCUU^ half, respectively) for YBX1 binding, confirmed that the 5′-tRNA^LysCUU^ half effectively displaces YBX1 from LL588.

tRNA halves are among the most abundant sncRNAs in the cellular transcriptome. In macrophages, the abundance of 5′-tRNA^HisGUG^ half is over 136-fold greater than that of the most abundant miRNA, miR-150 [4]. The 5′-tRNA^GlyGCC^ half is present at concentrations of 13–18 fmol per 1 μg of total RNA across various mouse tissues, whereas the “total” miRNA concentration ranges only from 0.005 to 8.8 fmol [9]. This study presents the first example of how these abundant molecules can post-transcriptionally regulate gene expression by impacting mRNA stability. Our findings suggest that YBX1 stabilizes p21 mRNA, a negative regulator of the cell cycle, by directly binding to 5′-tRNA^LysCUU^ half-like sequences, specifically LL588, in the 3′-UTR. When sex hormone signaling pathways induce the abundant accumulation of tRNA halves, the 5′-tRNA^LysCUU^ half acts as a destabilizer of p21 mRNA by trapping YBX1 from p21 mRNA, potentially serving as the molecular mechanism underlying the tRNA half-mediated promotion of the cell cycle and cell proliferation (S4 Fig). Unlike tRNA halves induced by stress or infection, sex hormone-dependent tRNA halves are constitutively expressed in hormone-dependent cancer cells. These cancer cells may utilize 5′-tRNA halves to promote cell growth by continuously sequestering YBX1, thereby maintaining low p21 levels. The mechanisms underlying the constitutive presence of 5′-tRNA halves in these cancer cells warrant further investigation. In addition, YBX1-interacting RNAs require a comprehensive exploration, such as via HITS-CLIP, to determine whether other 5′-tRNA halves and mRNAs are regulated through similar mechanisms. Because 5′-tRNA halves contain a 2′,3′-cyclic phosphate (cP) [5], and thus were not captured in previous CLIP studies for YBX1 [14], future analyses should employ modified CLIP protocols—such as inserting T4 polynucleotide kinase treatment to dephosphorylate the cP—for capturing YBX1-interacting RNAs comprehensively. There is over 3,000 mRNA-binding proteins that could potentially regulate mRNA stability [16]. Different 5′-tRNA halves may trap various different proteins to govern distinct mRNA profiles. Unlike the case of seed regions of the 3′-tRNA fragments acting as miRNAs [17], the major species of 5′-tRNA halves exhibit broad sequence variations [4,5,9,10]. mRNAs containing sequences similar to those of various 5′-tRNA halves might be regulated by proteins that bind to the respective 5′-tRNA half species. Further research is required to elucidate the potential presence of the widespread regulatory networks affecting mRNA stability, orchestrated by diverse species of 5′-tRNA halves and their interacting proteins.

## Materials and methods

### Cell culture, RNAi KD, plasmid transfection, and OE of tRNA halves

LNCaP cells were cultured in Improved MEM (Richter’s Mod.) with L-glutamine (Corning) containing 5% FBS. RNAi KD of 5′-tRNA halves and YBX1 was performed as described previously [5] using the siRNAs whose sequences of the sense strand with 3′-overhangs are the following: 5′-tRNA^LysCUU^ half (si5′-LysCUU), 5′-AGCUCAGUCGGUAGAGCAUUU-3′; 5′-tRNA^LysCUU^ half (si5′-LysCUU2), 5′-GCUAGCUCAGUCGGUAGAGUU-3′; 5′-tRNA^LysCUU^ half (si5′-LysCUU3), 5′-GUCGGUAGAGCAUGGGACUUU-3′; 3′-tRNA^LysCUU^ half, 5′-CUCAGGGUCGUGGGUUCGAUU-3′; and YBX1, 5′-UGACACCAAGGAAGAUGUAUU-3′. The ON-TARGETplus Non-targeting siRNA #2 (#D-001810–02; Dharmacon) was used as a negative control. The LNCaP cells were transfected with each siRNA (20 nM for 5′- and 3′-tRNA^LysCUU^ halves and 50 nM for YBX1) using the reverse transfection method with Lipofectamine RNAiMAX Transfection Reagent (Invitrogen). For the OE of YBX1, we cloned YBX1 CDS, amplified from LNCaP total RNA, into a pIRES2-ZsGreen1 (Clontech). The plasmid was transfected into LNCaP cells using Lipofectamine 2000 Transfection Reagent (Invitrogen). After 72 h, G418 (Enzo Life Sciences) was added into the culturing medium for selection, followed by isolation of the YBX1 OE cells using BD FACSAria II, SORP (BD Biosciences). For the OE of tRNA halves, 5′-tRNA^LysCUU^ half (S1 Table), 3′-tRNA^LysCUU^ half (S1 Table), and control RNA derived from Rluc (5′-P-GGGAGGCAAGCCCGACGUCGUCCAGAUUGUCCGC-3′) [18] were synthesized by an in vitro reaction using T7 RNA polymerase (New England Biolabs) as described previously [4,19]. The LNCaP cells were transfected with 100 nM of each RNA using the reverse transfection method with Lipofectamine RNAiMAX Transfection Reagent (Invitrogen).

### Cell proliferation assay

LNCaP cells were plated in a 96-well cell culture plate. At 24, 48, 72, 96, and 120 h after siRNA transfection, the cells were counted using hemocytometer.

### PI flow cytometric assay

To analyze cell cycle arrest, PI flow cytometric assay was performed as described previously [20]. LNCaP cells were fixed by 70% ethanol for 30 min at −20 °C, followed by treatment with 2 mg/ml of RNase A (Thermo Scientific) for 20 min at 37 °C. Subsequently, the cells were stained with 20 μg/ml of PI for 30 min on ice, washed with PBS, and then subjected to the analyses using LSR II Flow Cytometer (BD Biosciences) at the Flow Cytometry Facility of the Sidney Kimmel Cancer Center at Thomas Jefferson University. The data analysis of 10,000 cells using FACSDiva software (BD Biosciences) was used for Fig 1C.

### Cell fractionation

Nuclear fraction of LNCaP cells was prepared by a standard method using hypotonic buffer. Briefly, approximately 25 μl of cell pellets were suspended in 400 μl of hypotonic buffer (20 mM Tris-HCl [pH 8.0], 10 mM KCl, 1 mM DTT, and 0.5 mM PMSF) and incubated on ice to swell the cells. Then 30 μl of 10% Triton X-100 was added to the cell suspension, followed by a weak vortexing for 30 s for the disruption of cell membrane. After centrifugation at 1,200 × *g* for 1 min, the obtained pellet (the crude nuclear fraction) was resuspended in 800 μl of the hypotonic buffer containing 0.5% Triton X-100, followed by additional centrifugation to obtain the nuclear fraction as a pellet. Cytosolic and mitochondrial fractions were prepared by using Qproteome Mitochondria Isolation Kit (Qiagen) with the following modifications. The kit-separated cytosolic fraction was subjected to centrifuge at 21,130 × *g* for 10 min, and the resultant supernatant was used as a cytosolic fraction. For mitochondrial fraction, the kit-separated mitochondrial fraction was treated with 0.1 mg/ml RNase A for 20 min on ice to remove RNAs attaching to mitochondrial surface.

### mRNA microarray

At 72 h after siRNA transfection for KD of 5′- or 3′-tRNA halves, total RNA from the cells was isolated using TRIsure (Bioline) and subjected to microarray analysis using Agilent’s Human Gene Expression 4 × 44K slide (MOgene). Two-color microarray determined the relative abundance of mRNAs between control and each tRNA half KD samples. Among the 19,699 gene spots with >20 raw signal counts (out of total 32,163 gene spots), Entrez or Ensembl gene IDs were identified for 19,557 gene spots. The gene IDs and their fold-change values in each KD experiment were subjected to statistical enrichment analysis on PANTHER Classification System [21]. The microarray data sets are available in the NCBI Sequence Read Archive under accession number GSE245843.

### Standard RT-qPCR for RNA quantification

Total RNA from the cells was treated with RQ1 DNase (Promega) and subjected to reverse transcription using RevertAid Reverse Transcriptase (Thermo Scientific) and a gene-specific reverse primer. The synthesized cDNAs were then subjected to PCR using SYBR Green PCR Master Mix (Applied Biosystems) and forward and reverse primers on the StepOnePlus Real-Time PCR System (Applied Biosystems). The expression levels were normalized to those of 5S rRNA. Sequences of the primers used are the following: p21 mRNA Forward 5′-AGCAGAGGAAGACCATGTGGA-3′, Reverse 5′-AGTGGTAGAAATCTGTCATGCTG-3′; p21 mRNA for crosslinking and immunoprecipitation experiment (described below) Forward 5′-CAAACGCCGGCTGATCTTCTC-3′, Reverse 5′-CAGGGTATGTACATGAGGAGGTG-3′; pre-p21 Forward 5′-CCACTGTCTTCCTCAGTTGG-3′, Reverse 5′-CCTCTTGGAGAAGATCAGCC-3′; YBX1 Forward 5′-CTCCATCTCCTACACTGCG-3′, Reverse 5′-GCGGGGACAAGAAGGTCAT-3′; GAPDH Forward 5′-GTCTTCACCACCATGGAGAAG-3′, Reverse 5′-ATGATCTTGAGGCTGTTGTCAT-3′; RPLP0 Forward 5′-CTATCATCAACGGGTACAAACGAG-3′, Reverse 5′-CAGATGGATCAGCCAAGAAGG-3′; U6 snRNA Forward 5′-TCGCTTCGGCAGCACATATAC-3′, Reverse 5′-CGAATTTGCGTGTCATCCTTG-3′; mitochondrial 12S rRNA Forward 5′-CACTGCTGTTTCCCGTGGG-3′, Reverse 5′-GGTCCTAGCCTTTCTATTAGC-3′; 18S rRNA Forward 5′-GTTAATTCCGATAACGAACGAGAC-3′, Reverse 5′-GACATCTAAGGGCATCACAGACC-3′; and mature tRNA^LysCUU^ Forward 5′-GCCCGGCTAGCTCAG-3′, Reverse 5′-GCTCGAACCCACGACC-3′.

### TaqMan RT-qPCR for quantification of 5′-tRNA halves

The specific quantification of 5′-tRNA halves was performed by TaqMan RT-qPCR as described previously [4,5,22]. Briefly, total RNA was treated with T4 polynucleotide kinase (T4 PNK, New England Biolabs) to dephosphorylate the 3′-terminal cP, followed by ligation of 3′-adaptor (3′-AD: 5′-P-GAACACUGCGUUUGCUGGCUUUGAGAGUUCUACAGUCCGACGAUCddC-3′) using T4 RNA Ligase (T4 Rnl; Thermo Scientific). The ligated products were quantified by TaqMan RT-qPCR using the One Step PrimeScript RT-PCR Kit (TaKaRa) on the StepOnePlus Real-Time PCR System (Applied Biosystems). The expression levels were normalized to those of 5S rRNA. The sequences of the primers and TaqMan probes are the following: 5′-tRNA^LysCUU^ half Forward 5′-GCCCGGCTAGCTCAG-3′, Reverse 5′-GATCGTCGGACTGTAGAACTC-3′, TaqMan/56FAM/AGAGCATGG/ZEN/GACTCGAACACTG/3IABkFQ/; and 5S rRNA Forward 5′-TACGGCCATACCACCCTGAAC-3′, Reverse 5′-GATCGTCGGACTGTAGAACTC-3′, TaqMan/56-FAM/CGGGTGCTG/ZEN/TAGGCTTTGAACACTGCGTT/3IABkFQ/.

### Western blot

Western blots were performed as described previously [22]. The following antibodies were used: anti-p21 (Sigma–Aldrich, OP64), anti-GAPDH (Cell signaling, 2,118), anti-YB1 (Abcam, ab12148), anti-Flag (Sigma–Aldrich, F3165), anti-Histone H2B (Cell signaling, 8135S), and anti-β-Tubulin (Developmental Studies Hybridoma Bank, E7).

### Pull-down experiment and mass-spec protein identification

Total cell lysate or cytosolic fraction of LNCaP cells was pre-cleared with Streptavidin Sepharose High Performance (GE Healthcare Life Sciences) in a buffer containing 20 mM Tris-HCl (pH 7.5), 150 mM NaCl, 2.5 mM MgCl_2_, 0.5% NP-40, 0.1% Triton X-100, and cOmplete EDTA-free Protease Inhibitor Cocktail (Roche). The resultant lysate was incubated with 0.9–1.5 nmol of the biotinylated RNA (5′-tRNA^LysCUU^ half: 5′-P-GCCCGGCUAGCUCAGUCGGUAGAGCAUGGGACUC-Bio-3′; 3′-tRNA^LysCUU^ half: 5′-UUAAUCCCAGGGUCGUGGGUUCGAGCCCCACGUUGGGCGCCA-Bio-3′; control: 5′-P-GGGAGGCAAGCCCGACGUCGUCCAGAUUGUCCGC-Bio-3′) for 1 h at 4 °C, followed by addition of the Streptavidin Sepharose and further incubation for 2 h. After washing four times with the above-described buffer, more stringent wash was performed using the buffer with 400 mM NaCl. The RNA-binding proteins on the resultant beads were eluted with SDS–PAGE sample buffer and subjected to SDS–PAGE. The prominent bands were excised and analyzed by mass-spec at Taplin Mass Spectrometry Facility at Harvard Medical School.

### Determination of the absolute amounts of 5′-tRNA^LysCUU^ half and p21 mRNA

In vitro RNA synthesis of 5′-tRNA^LysCUU^ half was performed as described above, while full length of p21 mRNA was also synthesized in vitro and purified by MEGAclear Transcription Clean-Up Kit (Invitrogen). Each synthetic RNA (0.001–50 fmol) was mixed with 1 μg of *E.coli* total RNA and quantified using TaqMan or standard RT-qPCR as described above, yielding standard curves with excellent linearities. The absolute amounts of 5′-tRNA^LysCUU^ half and p21 mRNA were calculated based on the standard curves.

### UV crosslinking and immunoprecipitation

UV crosslinking and immunoprecipitation were performed using HITS-CLIP conditions as previously described [23,24]. YBX1-overexpressing LNCaP cells were washed with PBS (without Mg^2+^ and Ca^2+^) and then irradiated with UV (400 mJ/cm^2^) using a CL-1000 Ultraviolet Crosslinker (UVP). After the lysis of cells and subsequent treatment of the lysate with RQ1 DNase, immunoprecipitation of YBX1 was performed using Anti-FLAG M2 magnetic beads (Sigma–Aldrich), employing stringent conditions with 1× PMPG buffer (1× PBS without Mg^2+^/Ca^2+^ and 2% Empigen) supplemented with cOmplete EDTA-free Protease Inhibitor Cocktail (Roche) and RNasin Ribonuclease Inhibitor (Promega). Dynabeads Protein G beads (Invitrogen) were pre-incubated with normal mouse serum (NMS) and used in a negative control experiment.

### Luciferase assay

We cloned p21 CDS or 3′-UTR, amplified from LNCaP total RNA, into 3′-UTR of Rluc in psiCHECK-2 plasmid (Promega). The mutations and deletions in CDS and 3′-UTR (Fig 4D) were introduced by site-directed mutagenesis. The constructed plasmids were transfected into LNCaP cells using Lipofectamine 2000 Transfection Reagent (Invitrogen). After 72 h, luciferase activity was measured using the Dual-Luciferase Reporter Assay System (Promega) and the Synergy2 Multi-Mode Reader (BioTek). The Rluc activity was normalized by firefly luciferase (Fluc) activity. KD or OE of the 5′-tRNA^LysCUU^ half was performed 24 h after the luciferase plasmid transfection, and the luciferase activity was measured 72 h post-transfection.

### Pull-down competition assay

Control (5′-GCCCUAAUCCUAAGGGAGGCAAGCCCGACGUCGUCCAGAUUGUCCGCAACAAAGGCCCGC-3′), LL588 (5′-GCCCUAAUCCGCCCACAGGAAGCCUGCAGUCCUGGAAGCGCGAGGGCCUCAAAGGCCCGC-3′), and 5′-tRNA^LysCUU^ half were synthesized in vitro as described above. Biotinylation of the synthesized RNAs was performed as described previously [25]. Briefly, the RNAs were oxidized in a buffer containing 100 mM sodium acetate (pH 5.0), 10 mM NaIO_4_, and 10 mM MgCl_2_ for 1 h at room temperature in the dark, followed by purification using NAP 5 gel filtration column (GE Healthcare Life Sciences) and ethanol precipitation. The oxidized RNAs were then subjected to biotinylation by incubating in 5 nM Biotin (Long Arm) hydrazide (Vector), 100 mM sodium acetate (pH 5.0), and 10 mM MgCl_2_ overnight at room temperature in the dark. After NAP5 purification and ethanol precipitation, 0.9 nmol of the biotinylated RNA and LNCaP cytosolic fraction were incubated, followed by pull-down of interacting proteins as described above. For competition assay, the pull-down beads were incubated with the different amounts [×1 (0.9 nmol), × 5 (4.5 nmol), or ×10 (9 nmol)] of competitor RNA (without biotin) for 1h at room temperature in a buffer containing 20 mM Tris-HCl (pH 7.5), 150 mM NaCl, 2.5 mM MgCl_2_, 0.5% NP-40, and 0.1% Triton X-100. After washing the beads with the above buffer containing 400 mM NaCl, the beads-binding proteins were eluted with SDS sample buffer and subjected to western blot analysis.

### Expression and purification of recombinant YBX1 protein

The plasmid coding Flag-YBX1 described above was transfected into HEK293T cells using polyethylenimine. After 48 h, cells were harvested and resuspended in lysis buffer containing 50 mM Tris–HCl (pH 7.5), 300 mM NaCl, 2 mM EDTA, 0.1% Triton X-100, and cOmplete EDTA-free Protease Inhibitor Cocktail. Following sonication, the supernatant was incubated with ANTI-FLAG M2 Affinity Gel (Sigma–Aldrich), and the bound protein was eluted with 200 μg/ml 3X FLAG Peptide (Sigma–Aldrich).

### DRaCALA

DRaCALA was performed as described previously [15]. Briefly, 1.5 μM Flag-YBX1 and 2 nM ^32^P-labeled RNAs were mixed in a buffer containing 40 mM Tris-HCl (pH 7.5), 50 mM NaCl, 2.5 mM MgCl_2_, 2 mM DTT, and 0.01% Triton X-100, and incubated at 37 °C for 30 min. A 3 μl aliquot of the mixture was then spotted onto nitrocellulose and allowed to dry completely. Signals were detected using a Typhoon laser scanner (Cytiva) and the fraction bound was calculated as described previously [15]. For the competition assay using DRaCALA, 2 μM Flag-YBX1, 2 nM ^32^P-labeled RNA, and either 100 μM or 200 μM unlabeled RNA (as competitor) were mixed in the same buffer and incubated at 37 °C for 30 min. A 3 μl aliquot of the mixture was spotted onto nitrocellulose, and the signals were detected as described above.

## Supporting information

S1 Fig Scatterplot of microarray data. Scatterplot showing the correlation between the signal intensity of 5′-tRNA^LysCUU^ half KD and the control KD samples. Pink dots represent CDK inhibitors and p53 shown in Fig 2A. The blue line indicates a 2-fold change boundary relative to the black dashed line. The data underlying the graphs can be found in S1 Data.
(PDF)

S2 Fig 5′-tRNA^LysCUU^ half does not affect the luciferase activity of the LL588-lacking construct. The luciferase assay shown in Fig 4E was performed using LNCaP cells with either KD or OE of the 5′-tRNA^LysCUU^ half. In all bar graphs in the present study, error bars indicate mean ± SD of triplicate measurements (*P < 0.05, **P < 0.01, and ***P < 0.001; two-tailed *t* Test). The data underlying the graphs can be found in S1 Data.
(PDF)

S3 Fig Recombinant YBX1 exhibited strong binding to the 5′-tRNA^LysCUU^ half. **(A)** Purified full-length YBX1 protein visualized by SDS–PAGE. **(B)** Representative DRaCALA images showing YBX1 binding of ^32^P-labeled 5′-tRNA^LysCUU^ half, LL588, and 3′-tRNA^LysCUU^ half. The bar graph represents the relative amounts of each RNA bound to YBX1, calculated from the DRaCALA results. Raw images of **A and B** are located in S1 Raw images. The data underlying the graphs can be found in S1 Data.
(PDF)

S4 Fig Schematic representation of how the 5′-tRNA^LysCUU^ half promotes cell proliferation. (PDF)

S1 Table Sequences and unique IDs for the tRNA halves focused on in this study. (PDF)

S1 Data merical data underlying Fig 1A, 1B, and 1E; Fig 2A–2C and 2E; Fig 3A, 3E, 3G, and 3H; Fig 4A, 4B, 4E, and 4I; S1 Fig; S2 Fig and S3B. (XLSX)

S1 Raw images Original images contained in this manuscript, related to Fig 2D; Fig 3B–3D, and 3F; Fig 4B and 4G–4I; S3A Fig and S3B Fig. (PDF)

## Abbreviations

ANG,: angiogenin;
AR: androgen receptor
CDK: cyclin-dependent kinase
DRaCALA: differential radial capillary action of ligand assay
KD: knockdown
miRNAs: microRNAs
np: nucleotide position
OE: overexpression
PI: propidium iodide
sncRNAs: short non-coding RNAs
tRNA: transfer RNA
UTR,: untranslated region;
YBX1: Y-box binding protein 1
