# Supplementary material for: A hormone-dependent tRNA half promotes cell cycle progression via destabilization of p21 mRNA
Source: PLoS Biol. 2025 Jun 5;23(6):e3003194. doi: 10.1371/journal.pbio.3003194 (PMC12140204; doi:10.1371/journal.pbio.3003194)
Supplement: S1 Fig — Scatterplot showing the correlation between the signal intensity of 5′-tRNALysCUU half KD and the control KD samples. Pink dots represent CDK inhibitors and p53 shown in Fig 2A. The blue line indicates a 2-fold change boundary relative to the black dashed line. The data underlying the graphs can be found in S1 Data. (PDF) [file pbio.3003194.s001.pdf]

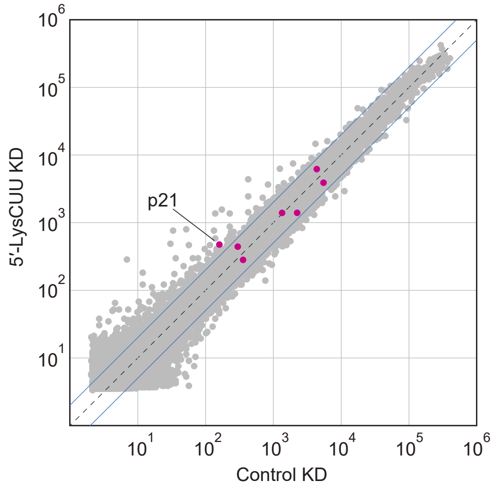

S1 Fig. Scatterplot of microarray data

Scatterplot showing the correlation between the signal intensity of 5′-tRNA^{LysCUU} half KD and the control KD samples. Pink dots represent CDK inhibitors and p53 shown in Fig. 2A. The blue line indicates a 2-fold change boundary relative to the black dashed line.
