# Supplementary material for: A hormone-dependent tRNA half promotes cell cycle progression via destabilization of p21 mRNA
Source: PLoS Biol. 2025 Jun 5;23(6):e3003194. doi: 10.1371/journal.pbio.3003194 (PMC12140204; doi:10.1371/journal.pbio.3003194)
Supplement: S2 Fig — The luciferase assay shown in Fig 4E was performed using LNCaP cells with either KD or OE of the 5′-tRNALysCUU half. In all bar graphs in the present study, error bars indicate mean ± SD of triplicate measurements (*P < 0.05, **P < 0.01, and ***P < 0.001; two-tailed t Test). The data underlying the graphs can be found in S1 Data. (PDF) [file pbio.3003194.s002.pdf]

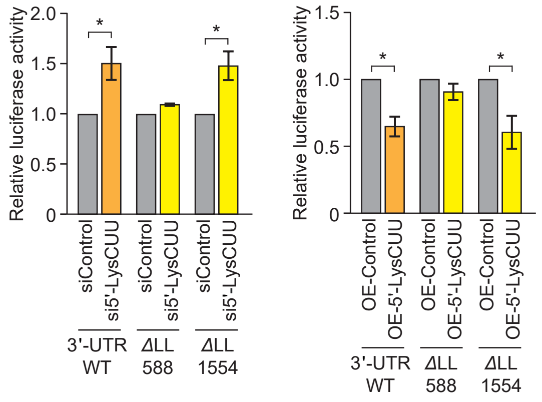

S2 Fig. 5′-tRNA^{LysCUU} half does not affect the luciferase activity of the LL588-lacking construct

The luciferase assay shown in Fig. 4E was performed using LNCaP cells with either KD or OE of the 5′-tRNA^{LysCUU} half. In all bar graphs in the present study, error bars indicate mean ± SD of triplicate measurements (**P* < 0.05, ***P* < 0.01, and ****P* < 0.001; two-tailed *t*-Test).
