# Supplementary material for: A hormone-dependent tRNA half promotes cell cycle progression via destabilization of p21 mRNA
Source: PLoS Biol. 2025 Jun 5;23(6):e3003194. doi: 10.1371/journal.pbio.3003194 (PMC12140204; doi:10.1371/journal.pbio.3003194)
Supplement: S3 Fig — (A) Purified full-length YBX1 protein visualized by SDS–PAGE. (B) Representative DRaCALA images showing YBX1 binding of 32P-labeled 5′-tRNALysCUU half, LL588, and 3′-tRNALysCUU half. The bar graph represents the relative amounts of each RNA bound to YBX1, calculated from the DRaCALA results. Raw images of A and B are located in S1 Raw images. The data underlying the graphs can be found in S1 Data. (PDF) [file pbio.3003194.s003.pdf]

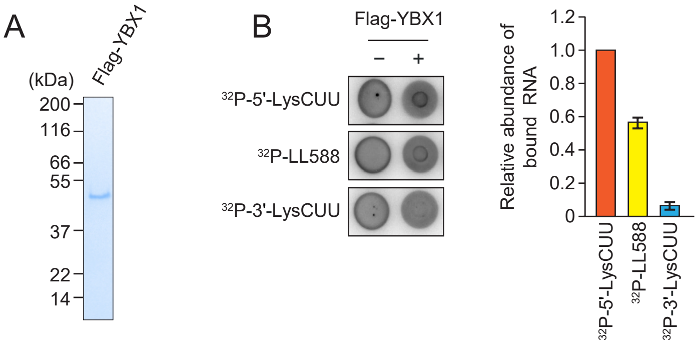

S3 Fig. Recombinant YBX1 exhibited strong binding to the 5′-tRNALysCUU half

(A) Purified full-length YBX1 protein visualized by SDS-PAGE.

(B) Representative DRaCALA images showing YBX1 binding of ^{32}P-labeled 5′-tRNA^{LysCUU} half, LL588, and 3′-tRNA^{LysCUU} half. The bar graph represents the relative amounts of each RNA bound to YBX1, calculated from the DRaCALA results. Raw images of A and B are located in S1 Raw Images.
