# Supplementary material for: A hormone-dependent tRNA half promotes cell cycle progression via destabilization of p21 mRNA
Source: PLoS Biol. 2025 Jun 5;23(6):e3003194. doi: 10.1371/journal.pbio.3003194 (PMC12140204; doi:10.1371/journal.pbio.3003194)
Supplement: S4 Fig — (PDF) [file pbio.3003194.s004.pdf]

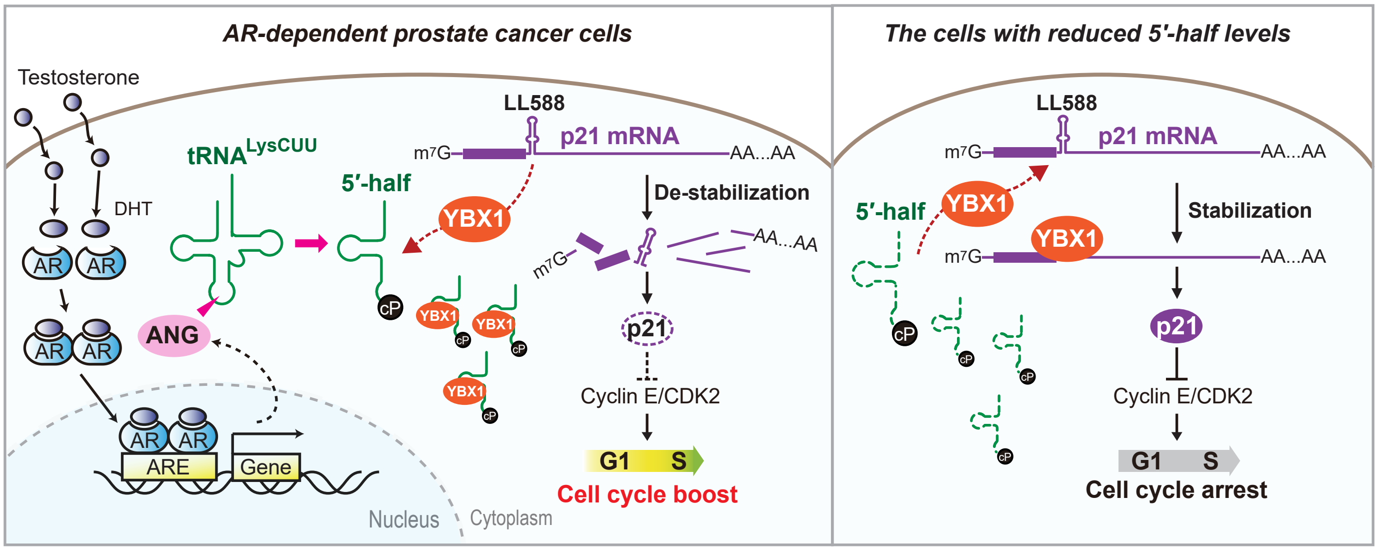

S4 Fig. Schematic representation of how the 5′-tRNA^{LysCUU} half promotes cell proliferation
