# Supplementary material for: A hormone-dependent tRNA half promotes cell cycle progression via destabilization of p21 mRNA
Source: PLoS Biol. 2025 Jun 5;23(6):e3003194. doi: 10.1371/journal.pbio.3003194 (PMC12140204; doi:10.1371/journal.pbio.3003194)
Supplement: S1 Table — (PDF) [file pbio.3003194.s005.pdf]

S1 Table. Sequences and unique IDs for the tRNA halves focused on in this study

RNA	Sequence (5′–3′)	tDRname	Licence plate
5′-tRNALysCUU half	GCCCGGCUAGCUCAGUCGGUAGAGCAUGGGACUC	tDR-1:34-Lys-CTT-1-M2	tRF-34-PSQP4PW3FJIKE5
3′-tRNALysCUU half	UUAAUCCCAGGGUCGUGGGUUCGAGCCCCACGUUGGGCGCCA	tDR-35:76-Lys-CTT-1	tRF-42-YEKPRS93W0K6MY6V2
