# Supplementary figures and images for: A hormone-dependent tRNA half promotes cell cycle progression via destabilization of p21 mRNA
Source: PLoS Biol. 2025 Jun 5;23(6):e3003194. doi: 10.1371/journal.pbio.3003194 (PMC12140204; doi:10.1371/journal.pbio.3003194)

Fig. 2D

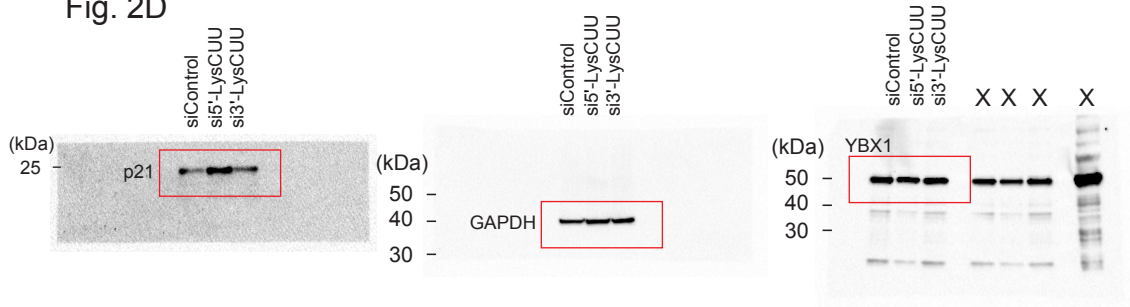

Fig. 3B

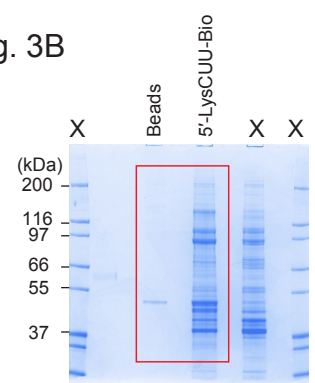

Fig. 3C

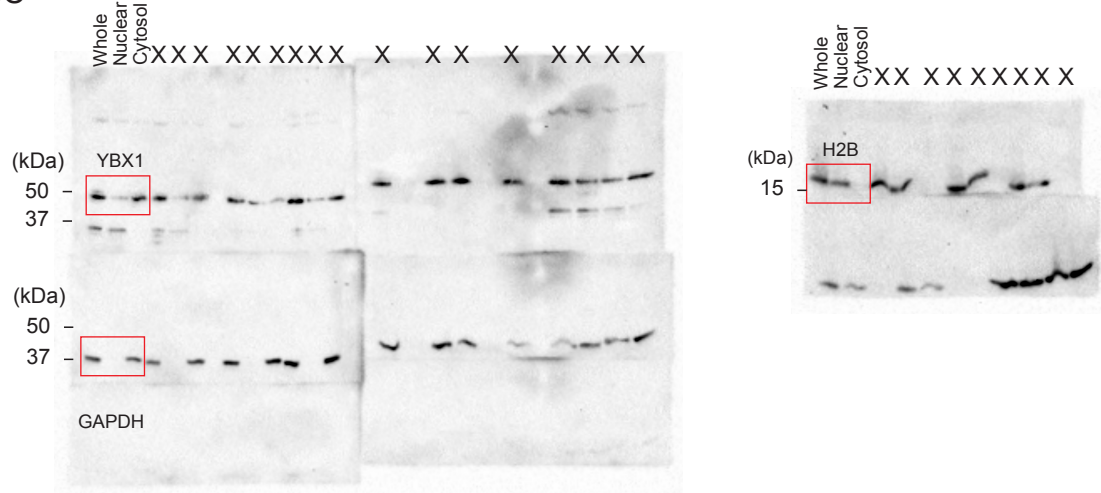

Fig. 3D

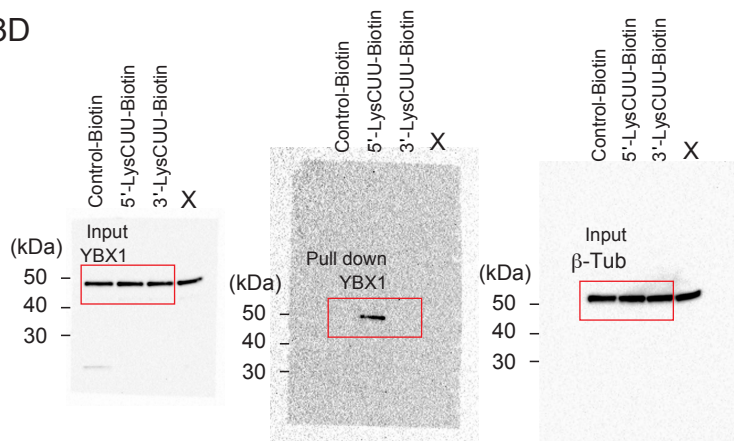

Fig. 3F

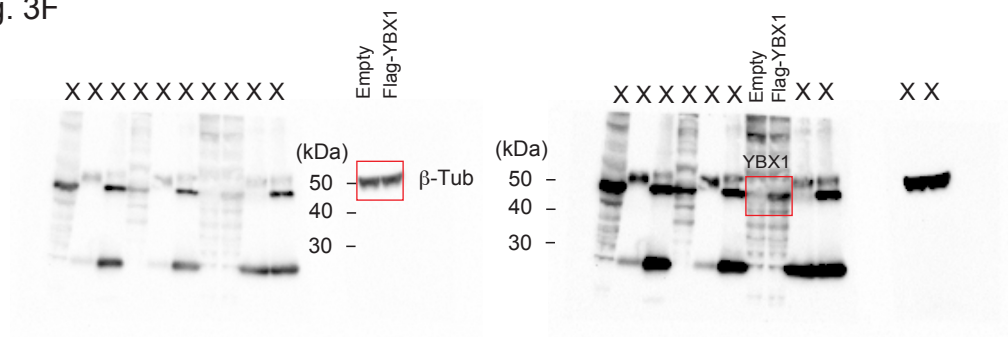

Fig. 4B

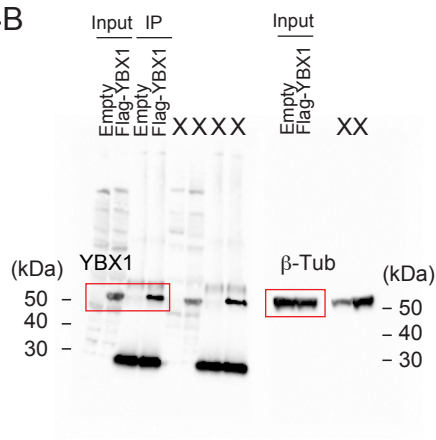

Fig. 4G

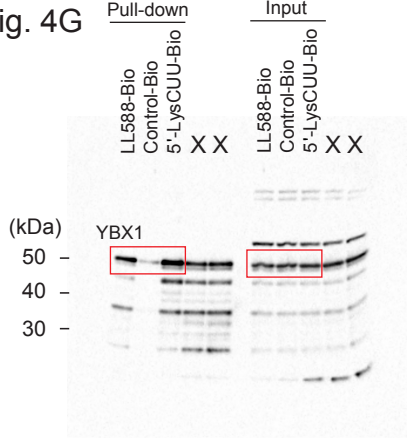

Fig. 4H

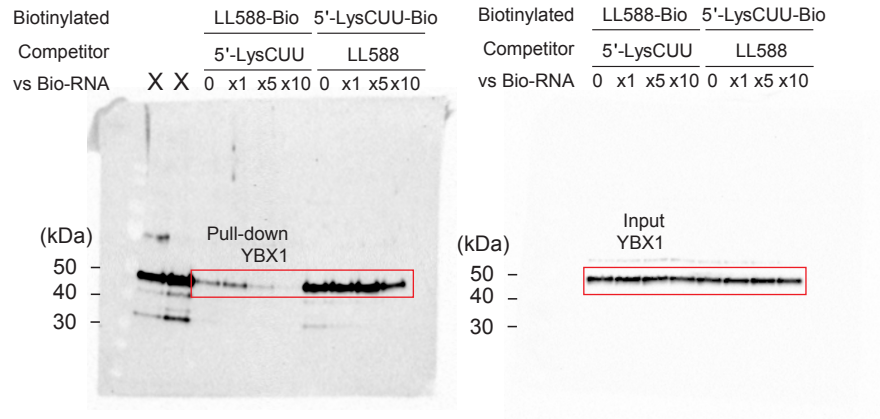

Fig. 4I

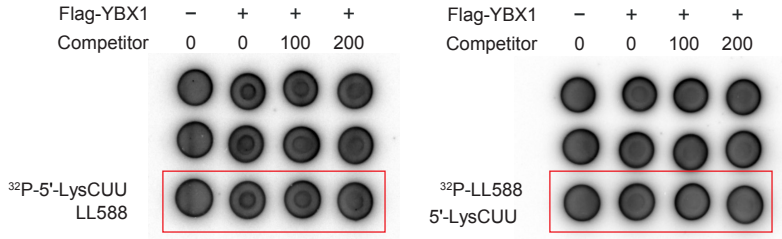

S3 Fig. A

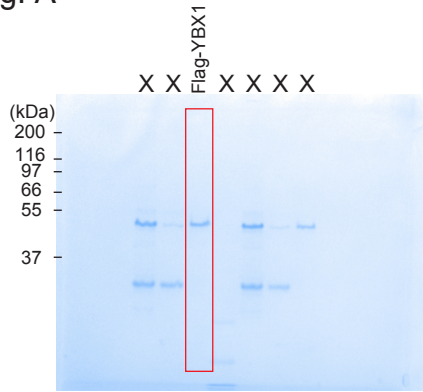

S3 Fig. B

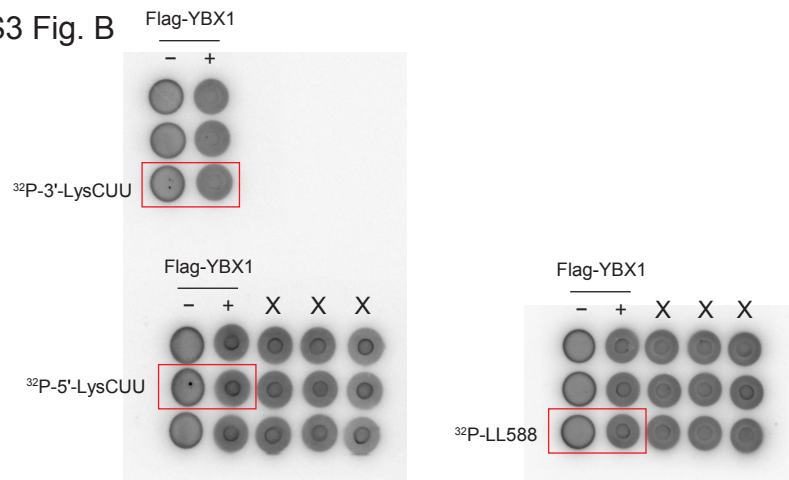

Supplement: S1 Raw images — (PDF) [file pbio.3003194.s007.pdf]
